# Osteochondral Injury, Management and Tissue Engineering Approaches

**DOI:** 10.3389/fcell.2020.580868

**Published:** 2020-11-04

**Authors:** George Jacob, Kazunori Shimomura, Norimasa Nakamura

**Affiliations:** ^1^Department of Orthopedic Surgery, Osaka University Graduate School of Medicine, Osaka, Japan; ^2^Department of Orthopedics, Tejasvini Hospital, Mangalore, India; ^3^Institute for Medical Science in Sports, Osaka Health Science University, Osaka, Japan; ^4^Global Center for Medical Engineering and Informatics, Osaka University, Osaka, Japan

**Keywords:** osteochondral repair, Tissue Engineering and Regenerative Medicine, articular cartilage, multiphasic scaffold, Mesenchymal stem cell

## Abstract

Osteochondral lesions (OL) are a common clinical problem for orthopedic surgeons worldwide and are associated with multiple clinical scenarios ranging from trauma to osteonecrosis. OL vary from chondral lesions in that they involve the subchondral bone and chondral surface, making their management more complex than an isolated chondral injury. Subchondral bone involvement allows for a natural healing response from the body as marrow elements are able to come into contact with the defect site. However, this repair is inadequate resulting in fibrous scar tissue. The second differentiating feature of OL is that damage to the subchondral bone has deleterious effects on the mechanical strength and nutritive capabilities to the chondral joint surface. The clinical solution must, therefore, address both the articular cartilage as well as the subchondral bone beneath it to restore and preserve joint health. Both cartilage and subchondral bone have distinctive functional requirements and therefore their physical and biological characteristics are very much dissimilar, yet they must work together as one unit for ideal joint functioning. In the past, the obvious solution was autologous graft transfer, where an osteochondral bone plug was harvested from a non-weight bearing portion of the joint and implanted into the defect site. Allografts have been utilized similarly to eliminate the donor site morbidity associated with autologous techniques and overall results have been good but both techniques have their drawbacks and limitations. Tissue engineering has thus been an attractive option to create multiphasic scaffolds and implants. Biphasic and triphasic implants have been under explored and have both a chondral and subchondral component with an interface between the two to deliver an implant which is biocompatible and emulates the osteochondral unit as a whole. It has been a challenge to develop such implants and many manufacturing techniques have been utilized to bring together two unalike materials and combine them with cellular therapies. We summarize the functions of the osteochondral unit and describe the currently available management techniques under study.

## Introduction

Osteochondral lesions (OL) are a morphological finding as a result of an acute trauma or occur due to osteochondritis dissecans, osteoarthritis (OA), subchondral insufficiency fractures or osteonecrosis (Gorbachova et al., 2018). OLs pose a difficult clinical situation for joint preservation surgeons as they extend beyond the articular cartilage into the subchondral bone and marrow (Gorbachova et al., 2018). The additional injury to the underlying structural support system along with the articular cartilage demands a more comprehensive clinical solution.

Due to the avascular nature of cartilage tissue, it is beyond the reach of reparative growth factors and cells, therefore not fortunate to follow normal tissue injury response (Temenoff and Mikos, 2000; Sophia Fox et al., 2009). The natural healing of OLs vary from that of chondral lesions due to their subchondral extension resulting in spontaneous cellular repair (Charalambous, 2014). This natural response leads to the formation of unsatisfactory fibrocartilage and the articular surface degenerates over time (Ochi et al., 2001) progressing toward OA. OL treatment strategies must aim to address both the subchondral bone and chondral surface above it (Gomoll et al., 2010). Current techniques include autologous chondrocyte implantation (ACI), osteochondral grafting and a combination of ACI and grafting, depending on the lesion. More recent treatments have employed tissue engineering and stem cell therapies using biphasic and triphasic scaffolds to provide effective osteochondral repair. We aim to focus on the osteochondral unit, its management and new emerging technologies for OL treatment.

## Anatomy of Cartilage and Subchondral Bone

The osteochondral unit consists of a articular chondral component and a deeper subchondral bone component (Madry et al., 2010). Cartilage is composed mainly of a dense extracellular matrix (ECM) made up of water, type II collagen, and proteoglycans (Buckwalter and Mankin, 1997; Keeney and Pandit, 2009). Within the cartilage tissue lies specialized cells known as chondrocytes (Sophia Fox et al., 2009). Cartilage is distinct in that it is completely devoid of blood vessels, lymphatics, and nerves (Buckwalter and Mankin, 1997; Sofat et al., 2011). It is divided into four zones, the superficial, the middle, the deep, and the calcified cartilage zone. Each zone having a unique cell orientation, collagen fiber arrangement and ECM composition allowing it to fulfill different biomechanical functions. For example, the superficial zone serves to protect the deeper zones from shear forces while the deeper zones are better arranged to counter compressive forces (Sophia Fox et al., 2009).

**FIGURE 1:**
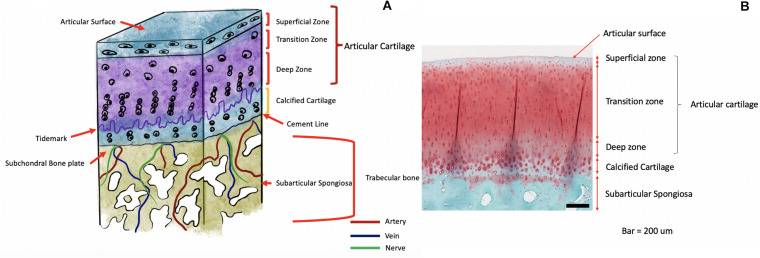
**(A)** Schematic Illustration of the anatomy of the osteochondral unit. **(B)** Histologic image of the osteochondral unit.

The deepest tissue of the osteochondral unit is the subchondral bone. Bone consists mainly of hydroxyapatite (HA) and type I collagen which contribute to the strength and stiffness of bone tissue (Yang and Temenoff, 2009; Arvidson et al., 2011). The subchondral bone region consists of thick plates joined together to form a subchondral bone plate below which is the subarticular spongiosa (Lopa and Madry, 2014). Separating the cartilage tissue and the bone is a complex junction known as the osteochondral junction also referred to by some authors as the chondro-osseous junction. It consists of the deepest zone of uncalcified cartilage, the tidemark, a layer of calcified cartilage, a thin line known as the cement line and beyond this the subchondral bone (Lyons et al., 2006). It is the tidemark that separates the non-calcified and calcified layer of cartilage from each other as a histologic wavy boundary up to 10 μm in thickness (Gannon and Sokoloff, 1999). Calcified cartilage has a lower mechanical strength than the bone below it (Mente and Lewis, 1994), however, a few unique features aid in better integration between the two layers (Nooeaid et al., 2012). Such as prolonged extensions of uncalcified cartilage extending through the calcified layer to abut the subchondral bone but not beyond it (Hunter et al., 2009). Also, the wavy nature of the tidemark and its vertically oriented fibers (Oegema et al., 1997). The junction is not impermeable (Madry et al., 2010) and a large number of arteries, veins, and nerves (Pan et al., 2009) send branches through minute canals within the subchondral plate into the calcified cartilage. This is the route by which nutrients are brought to the articular cartilage and a homeostatic environment maintained. In the setting of OA, there is the loss of articular cartilage, subchondral thickening, and formation of osteophytes (Haverkamp et al., 2011; Loeser et al., 2012) leading to loss of the normal biochemical and biomechanical processes Figure 1. Illustrates the cross-sectional anatomy of the osteochondral unit.

## Biomechanics of the Osteochondral Unit

The osteochondral unit performs several functions with each layer of the tissue having a specific and some overlapping roles. The chondral and subchondral tissues are separated by a chondro-osseous junction allowing them to work together to help the entire osteochondral unit accomplish its responsibilities in maintaining healthy joint homeostasis (Lyons et al., 2006).

The chondral layer being the most superficial layer of the osteochondral unit is subjected to a greater number of force vectors. As with most of the osteochondral unit, the chondral layer must withstand compressive forces but in addition to this, it must also counter friction and shear forces generated cyclically during normal joint articulation. Chondral tissue is best described as being biphasic as it demonstrated features of both a fluid and solid phase substance. Water and inorganic ions such as sodium, potassium, calcium and chloride are responsible for its fluid phase and ECM for its solid phase (Sophia Fox et al., 2009). With the presence of negatively charged proteoglycans (Ghadially, 1981) and the porous permeable ECM (Mow et al., 1984; Ateshian et al., 1997), interstitial fluids can move in and out of the tissue with the increasing and decreasing joint forces (Maroudas and Bullough, 1968; Mow et al., 1984; Frank and Grodzinsky, 1987). This summarizes the flow-dependent mechanism which allows for the chondral tissue to exhibit a biphasic viscoelastic behavior (Mow et al., 1980). The flow independent mechanism is brought about by the viscoelastic behavior of the collagen-proteoglycan matrix (Hayes and Bodine, 1978; Woo et al., 1987). As the forces increase on the chondral tissue the tissue becomes stiffer and more resistant to the forces applied due to these mechanisms.

The osteochondral junction is an integral region in the osteochondral unit allowing for communication between the lower subchondral bone and the upper chondral surface. This region encompasses arteries, nerves, and veins that extend from the subchondral bone up to the calcified cartilage, where nutrient exchange is facilitated (Honner and Thompson, 1971). It is also responsible for mineralization, directing cells into various types of chondrocytes. The calcified cartilage layer interdigitates with the subchondral bone and contains chondrocytes embedded within a mineralized ECM. These features help in giving it a high stiffness to anchor the cartilage to the subchondral bone below (Mente and Lewis, 1994).

The subchondral bone consists of impermeable compact bone with many penetrating vascular canals allowing it to play a role in both strength and nutrition to the tissues above it (Duncan et al., 1987; Aigner and Dudhia, 1997; Imhof et al., 2000). Sensory neurons innervate the subchondral bone region and provide nociception (Lepage et al., 2019). The deeper layers of the subchondral bone consist of trabecular bone which can absorb and dissipate the forces applied across the joint (Stewart and Kawcak, 2018) Figure 2. Summarizes the various specific roles of each layer of the osteochondral unit.

**FIGURE 2:**
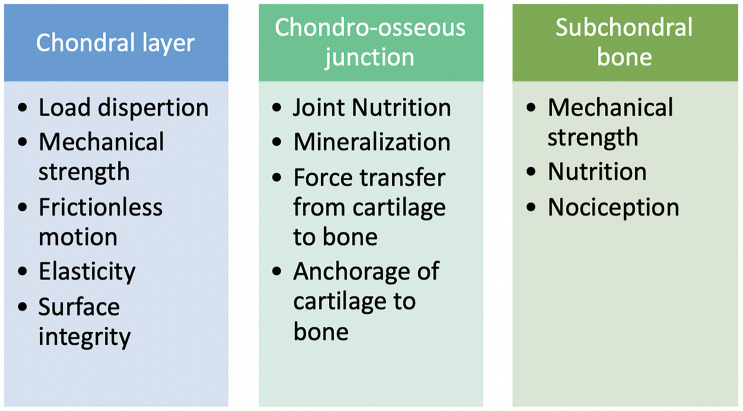
List of functional roles each layer of the osteochondral unit performs.

## Diagnosis of an Osteochondral Lesion

Clinical diagnosis of an OL can be elusive, when relying purely on patient complaints and clinical examination. Common complaints may or may not include an episode of trauma, however, there usually will be complaints of pain, swelling, crepitus, and possible history of knee locking. An examination may reveal joint line tenderness depending on the OL location and with associated injuries special meniscal and ligament tests may be positive. Older clinical tests for OLs such as Wilson’s test seem to not be of diagnostic value (Conrad and Stanitski, 2003).

Radiological diagnosis of an OL can be done by X-ray and computed tomography, but magnetic resonance imaging (MRI) has shown to be more useful especially in earlier stages of OL development. It can also detect earlier subchondral bone changes predisposing to OLs (Gorbachova et al., 2018). Thus, MRI with cartilage mapping software and sequences are the gold standard for radiologic evaluation of joint surfaces (Lepage et al., 2019). MRI has an important advantage over arthroscopy in that the status of the subchondral can be studied. MRI is also non-invasive and functions without the need of ionizing radiation and therefore well suited for OL evaluation. MRI studies allow for determination of lesion size, location, presence of bone marrow lesions, fracture lines, and subchondral plate deformities. These features can be used to make a diagnosis of the primary causes of the OL. Newer less invasive diagnostic arthroscopic techniques are being introduced and may be a useful tool in the outpatient clinic such as needle arthroscopy (McMillan et al., 2017) but can still only visualize the superficial chondral surface. It is also important to note that the clinical symptoms of an OL and diagnostic imaging may not correlate (Guermazi et al., 2012) making treatment decisions less straightforward. Additionally, it is important to diagnose the cause and chronicity of an OL. Acute traumatic lesions are a simpler clinical scenario while older more chronic lesions result in global degeneration of the involved joint. This eventually results in negative biochemical and biomechanical changes in the joint where most repair and regenerative treatments are no longer options and patients may have to consider other treatments such as arthroplasty.

## Repair Techniques

### Osteochondral Fragment Fixation

In certain acute traumatic OLs, there may be a large osteochondral fragment present within the joint that can be reduced and fixed back into the defect site. This is usually limited by the time from injury and the integrity and size of the fragment. Numerous fixation techniques have been described such as screws (Thomson, 1987; Herring et al., 2019), metal/bioabsorbable pins (Hirsch and Boman, 1998; Gkiokas et al., 2012), fibrin glue (Jeuken et al., 2019), and sutures (Vogel et al., 2020). Satisfactory osteochondral fragment union rates have been reported for each technique but there are some disadvantages and complications associated with each method (Gkiokas et al., 2012; Barrett et al., 2016; Herring et al., 2019) such as the requirement for second stage implant removal, tissue reactions, delayed degradation, and subchondral remodeling (Pascual-Garrido et al., 2009; Millington et al., 2010). Newer suture techniques have been described aiming to reduce the tissue reaction, implant footprint and requirement for implant removal (Vogel et al., 2020). Fixation in the case of a large acute osteochondral fragment should always be considered as the first option of treatment for an OL.

### Osteochondral Autologous Graft Transfer

Osteochondral autograft transfer (OAT) has been a popular technique since its first introduction by Matsusue et al. (1993). Here healthy articular cartilage and it’s underlying subchondral bone is harvested as a cylindrical plug, usually from the non-weight bearing region of the femoral trochlea. This cylinder is contoured to match the lesion site allowing for repair with smooth, healthy, mature, hyaline cartilage. The procedure can also be performed using a combination of multiple smaller diameter cylinders known as mosaicplasty. The advantages and disadvantages of OAT are summarized in Table 1. OAT has proven to be an attractive option in knee joint preservation surgery demonstrating good clinical outcomes and long term results, especially in lesions smaller than 2 cm^2^ (Gudas et al., 2012; Ulstein et al., 2014; Lynch et al., 2015; Pareek et al., 2016; Richter et al., 2016; Solheim et al., 2018). There is no difference in outcomes based on lesion location when assessing lesions on the femur, however, Bentley et al did report inferior outcomes when treating patellar lesions (Bentley et al., 2003). The main limitation for larger lesions is the amount of tissue required from the donor site and greater amounts of donor tissue lead to possibly increased donor site morbidity. It is in situations where larger amounts of donor tissue where a surgeon may have to resort to allogeneic graft options. A summary of the advantages and disadvantages of OATS can be found in Table 1.

**TABLE 1:** Summary of the advantages and disadvantages of OATS.

**Advantages**	**Disadvantages**
Mature hyaline cartilage	Limited quantity
No chance of immune response	Donor site morbidity
Immediate fill of lesion	Possibly >1 surgical site
No graft availability concerns	Limited lesion size
Addresses subchondral and chondral layer	

### Osteochondral Allograft Transplant

Osteochondral Allograft Transplant (OCA) is a popular technique used especially when dealing with larger OLs where an allograft is used for lesion restoration. This has all the advantages of OATS but with the added advantage of no donor site morbidity. OCA grafts can be fresh, fresh frozen or cryopreserved each having its effect on the chondrogenic viability of the cells within the graft. There is convincing data that patients tolerate allografting to the chondral component of the graft tissue with no immune response (Langer and Gross, 1974). This cannot be said for the subchondral and bony component which does elicit a strong immune response that increases with the size of graft tissue (Kandel et al., 1985; Stevenson et al., 1996). Patient selection is an important consideration in OCA transplantation and results have been superior in younger active patients (Krych et al., 2017; Nielsen et al., 2017). Factors such as age, sex, body mass index, and overall physical fitness play an important role in the prognosis of OCA (Sherman et al., 2014). OCA can be used for lesions >2 cm^2^ where marrow stimulation has been shown to have poor results. Over 2 cm^2^ cell-based techniques and OAT are options but cell-based techniques do not address subchondral bone pathologies and OAT has donor site morbidity. Therefore, OCA is indicated in lesions greater than 2 cm^2^ where autologous donor tissue is unavailable or insufficient. The indications of OCA have even extended to include the femoral hemicondyle, entire condyle, and even tibial plateau depending on the patient’s requirement (McCulloch et al., 2007; Williams et al., 2007; Levy et al., 2013). A summary of the advantages and disadvantages of OCA can be found in Table 2.

**TABLE 2:** Summary of the advantages and disadvantages of OCA.

**Advantages**	**Disadvantages**
Mature hyaline cartilage	Immunogenicity concerns
No limit to size of donor graft	Banked tissue therefore less cell viability
Immediate fill of lesion	Difficult to procure and store
Addresses subchondral and chondral layer	Additional expense
No donor site morbidity	Possible graft size mismatching

## Regenerative Techniques

**FIGURE 3:**
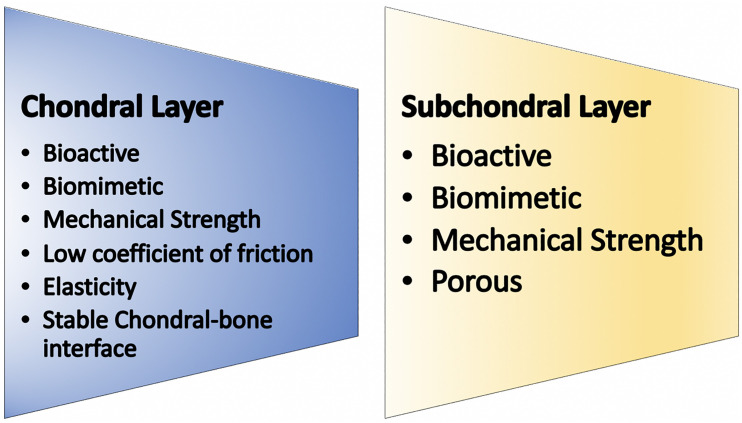
The ideal osteochondral unit.

Osteochondral unit regeneration is challenging owing to its complex anatomy and demanding functions. Both the chondral and subchondral layers must be regenerated for the regenerate to resemble native cartilage (Schek et al., 2004; Kon et al., 2011; Orth et al., 2013). In addition, both layers should integrate with the surrounding cartilage and bone tissue. Attempts have been made by developing biphasic scaffolds that have an osseous layer providing rigid, structural support incorporated to a more bioactive chondral layer into which cells may be seeded. It has been postulated that a triphasic scaffold with an intermediate layer between the chondral and bone layers would be beneficial and emulate the tidemark found in a native osteochondral unit (Marquass et al., 2010; Longley et al., 2018). This intermediate layer would have to mimic the osteochondral junction containing intricate networks of arteries and nerves with no currently available faultless biomaterial. Finally, the subchondral region would have to promote bony ingrowth from the surrounding tissue and have a low elastic modulus, providing strength to the entire construct. Figure 3 outlines the ideal requirements of a regenerated osteochondral unit.

### Cells

As mentioned earlier a regenerative osteochondral implant must be bioactive and the most important factor to achieve this is the addition of a cells. Various cell sources have been studied and employed such as embryonic stem cells (ESCs) and MSCs. MSCs have remained more popular given the ethical obstacles associated with ESCs (Lo and Parham, 2009). MSCs have been derived from a variety of tissues such as bone marrow (Mafi, 2011), adipose (Berebichez-Fridman et al., 2017), synovium (Sakaguchi et al., 2005), periosteum (de Mara et al., 2011), muscle (Jackson et al., 2010), dental pulp (Pierdomenico et al., 2005), and many more. More recently the discovery of the induced pluripotent stem cell (iPS cell) has made available a more easily accessible cell source with superior differentiation and proliferative potential (Takahashi and Yamanaka, 2006; Yu et al., 2007). Embryonic and induced pluripotent stem cells can differentiate into any of the three germ layers therefore along with having limitless proliferative potential and superior differentiation capacity they both pose a risk of teratoma formation (Tsumaki et al., 2015; Chijimatsu et al., 2017). Each MSC source has its own advantages and disadvantages. Concerning cell number harvest, adipose has shown the greatest yield while bone marrow the least (Baer and Geiger, 2012; Chahla et al., 2016). Synovium has demonstrated to have the most superior osteogenic and chondrogenic differentiation capacity compared to bone marrow and adipose, however, requires expansion when used in clinical applications (Sakaguchi et al., 2005). The high MSC yield in adipose tissue is beneficial as *in vitro* expansion has shown to have negative effects on cell homing (Sohni and Verfaillie, 2013).

Cells have been incorporated with tissue engineering for osteochondral regeneration with various techniques investigated till date. In the past, an autologous biopsy of chondrocytes and osteoblasts from the patient done during ACI was the most popular solution as obtaining the cells was easier, however, this did not result in sufficient cell numbers. At the expense of expanding the cells to increase the numbers in primary culture, the cells undergo dedifferentiation and lose their chondrogenic phenotype (Benya and Shaffer, 1982; Stewart et al., 2000; Schnabel et al., 2002; Darling and Athanasiou, 2005). Though ACI has been further improved over the years with the addition of collagen membranes and scaffolds, there is still no evidence that it is superior to other cartilage repair techniques (Samsudin and Kamarul, 2016). ACI also includes the higher cost of two surgeries and donor site morbidity. MSC therapies provide a solution in terms of not requiring a autologous articular cartilage biopsy along with providing pluripotent MSCs which are unlike already differentiated chondrocytes. MSCs can be be differentiated into chondrocytes by first isolating them from any of the aforementioned sources (Sundelacruz and Kaplan, 2009; Mafi, 2011; Nooeaid et al., 2012) or can be co-cultured with chondrocytes. This has shown to help maintain chondrocyte phenotype and characteristics (Hubka et al., 2014). It is worth mentioning that iPS cells have been studied for cartilage culture and maybe a promising source of stem cell going forward (Ko et al., 2014;Tsumaki et al., 2015). The most important advantage of using MSCs is that they are bioactive and therefore offer better incorporation with the body and can influence and mediate biological processes effectively. The cells exhibit paracrine functions (Hocking and Gibran, 2010; Barry and Murphy, 2013) which promote cell growth and have anti-inflammatory roles (Johnstone et al., 1998; Freyria and Mallein-Gerin, 2012; Ruiz et al., 2016). MSCs also stimulate endogenous cell recruitment and are involved with immunomodulation as well as secrete exosomes (Kanazawa et al., 2011; Zhang et al., 2020). MSC therapies are, however, considerably expensive and time consuming therefore acellular techniques are being explored (Dhollander et al., 2012; Joshi et al., 2012) though most preclinical data is in favor of cell-seeded scaffolds with a subchondral osteoinductive scaffold (Lopa and Madry, 2014). MSC treatments do, however, remain costly.

### Growth Factors

Growth factors are the most bioactive part of a regenerative process in that they control and initiate a host of cellular mechanisms which lead to superior chondrogenesis and cell proliferation. The most often utilized growth factors for chondrogenic differentiation are those in the TGF- β superfamily. This consists of bone morphogenic proteins (BMP-2,4,6, 7), cartilage-derived morphogenic proteins (CDMP-1,2), and transforming growth factor beta-1 (TGF-β). These factors are especially useful to stimulate chondrogenic differentiation, reverse dedifferentiation and encourage the production of ECM an essential component of chondral tissue. They also have an overall inhibitory effect on catabolic processes mediated by Interleukin-1,6,8 and matrix metalloproteinases (MMP; Salgado et al., 2004; Fortier et al., 2011; Re’Em et al., 2012; Tuan et al., 2013). Fibroblast growth factor (FGF) -2 and 18 play a prominent role in chondrogenic differentiation and seem to have different actions on MSCs and chondrocytes. FGF-2 and FGF-18 promote anabolic and reduce catabolic cell pathways which in turn lead to increased proteoglycan (PG) synthesis in MSCs (Cuevas et al., 1988; Stewart et al., 2007; Fortier et al., 2011). However, pre-clinical data has suggested FGF-2 to have deleterious effects on chondrocyte proliferation especially in higher doses by upregulation of MMP and reducing PG synthesis and increasing inflammation (Tuan et al., 2013). On the other hand FGF-18 has shown to promote chondrocyte proliferation (Ellsworth et al., 2002; Ellman et al., 2008). FGF-2 and TGF-β have been noted to work together to enhance cartilage ECM formation under culture conditions of chondrogenic cells and are essential for cartilage homeostasis (Huang et al., 2018). The FGF family does have a prominent effect on MSCs though possibly not as beneficial to chondrocyte metabolism. Another growth factor involved in cartilage synthesis is insulin-like growth factor (IGF-1) which supports the roles of TGF- β and BMP-7 and upregulates anabolic mechanisms and downregulates catabolism in the cells. IGF-1 has shown to promote chondrogenic differentiation in MSCs in a synergistic manner alongside TGF- β and BMP-7 (Loeser et al., 2003; Zhou et al., 2016). It has been noted that with decreased IGF-1 there is reduction in chondrocyte number and proteoglycan synthesis (Wei et al., 2017). Platelet derived growth factor (PGDF) is another growth factor which has a role in increasing chondrocyte proliferation and proteoglycan synthesis. PGDF has also shown to reduce IL- β1 levels which are known to cause chondral degradation (Schmidt et al., 2006).

Platelet rich plasma (PRP), autologous conditioned plasma and bone marrow concentrate are considered to be abundant in growth factors and have been used in clinical practice (Jacob et al., 2017). These therapies are manufactured by concentrating blood or bone marrow aspirate using a centrifugation process or specific company system to concentrate native growth factors present in the sample. As there are multiple types of growth factors with varying functional roles being concentrated in these injections both growth factors that promote chondrocyte metabolism and inhibit it are being concentrated (Rutgers et al., 2010). Literature has reported these blood derived products to not be beneficial in promoting chondrogenic differentiation of MSCs (Rutgers et al., 2010; Liou et al., 2018), however, some other studies have shown benefit (Frisbie et al., 2007; Mishra et al., 2009). The advantage of such therapies is the easy availability of autologous growth factors but the major disadvantage is the lack of standardization and determination of exact factor concentrations (Chahla et al., 2017).

### Emerging Techniques

Newer cell culture methods have been explored aiming to alter the cell microenvironment to improve cell differentiation and result in better quality regenerate synthesis (Vats et al., 2006). Three dimensional MSC cultures and scaffolding techniques have shown to be effective in improving cell proliferation (Estes and Guilak, 2011). High density cultures techniques such as micro mass and pellet cultures have demonstrated superior chondrocyte differentiation as the three-dimensional nature of the culture simulates a similar microenvironment to that of tissue during embryogenesis (Pelttari et al., 2008; Zhang et al., 2010). Other explored culture techniques have involved varying hydrostatic pressures, the addition of mechanical loading and use of low oxygen tensions. These variations can be brought about to the cell culture by using bioreactors aiming to replicate physiologic *in vivo* conditions. Various designs of bioreactors have been manufactured to produced compressive forces, shear forces and even dynamic cyclic loading of the cell culture (Angele et al., 2004; Campbell et al., 2006; Chen et al., 2018). Applying cyclical increasing hydrostatic pressures on MSC cultures has shown to enhance the production of cartilage matrix even in the absence of chondrogenic growth factors (Miyanishi et al., 2006; Puetzer et al., 2013). A large number of studies have reported improved chondrogenesis in cultures exposed to mechanical loading with dynamic, shear or compression forces (Huang et al., 2004; Mouw et al., 2007; Waldman et al., 2007; Villanueva et al., 2009). This emulates joint reaction forces and the additional mechanical stimulation on the chondrocytes results in better chondrogenic differentiation and matrix production. With this evidence it is reasonable to say that for improved chondrogenesis the cells require both growth factors and mechanical forces to bring about more physiological cellular responses.

## Scaffolds

### Chondral Layer

Synthetic polymers or natural biomaterial-based scaffolds are generally utilized for constructing the chondral component of the osteochondral unit. Though, recent reports have utilized scaffold-free implants as well. Because natural-based polymers are fabricated from materials that make up typical natural cellular environments, they may be ideal for cell proliferation with a reduced possibility of unfavorable reactions. Natural polymers may additionally be able to enhance cell proliferation and guide cellular differentiation to more desirable results (Mano and Reis, 2007; Nooeaid et al., 2012). In the process of procuring biocompatibility through these scaffolds they may lack mechanical vigor (Nooeaid et al., 2012). Commonly employed materials include chitosan, collagen, hyaluronic acid, and bio-based polymers (Shimomura et al., 2014b).

Bio-degradable synthetic scaffolds include poly (glycolic acid), poly (L-lactic acid), poly (D, L-lactic-co-glycolic acid), poly (caprolactone), and poly (ethylene glycol). These are superior to natural scaffolds in that their mechanical strength and crystallinity can be varied during manufacture along with the rate at which they undergo degradation (Gunatillake et al., 2003; Kundu et al., 2013). Furthermore, with newer techniques such as electrospinning and 3D printing, scaffold porosity, and shape are easily modifiable and constructed based on the requirement (Woodfield et al., 2005; Li et al., 2007; Thorvaldsson et al., 2008; Duan et al., 2014; Zhou et al., 2018). The major drawback of synthetic scaffolds is their poor bioactivity owing to their hydrophobic surfaces hindering cellular attachment (Bhattarai et al., 2006; Lee et al., 2006). These scaffolds may also be combined with growth factors (Morille et al., 2013; Reyes et al., 2014) and materials such as silica and alkalis to improve their bioactivity (Peña et al., 2006; Buchtová et al., 2013).

Extracellular matrix can provide a form of scaffolding to an osteochondral repair by providing some form of tissue architectural structure as well as bio signaling (Sutherland et al., 2015). Using chemical or physical methods cartilage ECM can be decellularized and then used to facilitate chondrogenic differentiation of MSCs (Cheng et al., 2009; Sutherland et al., 2015). Another method similar to an ECM is the use of a cell-derived matrix such as tissue-engineered construct (TEC) derived from synovial MSCs (Ando et al., 2007). TEC has favorable properties of being superiorly bioactive and highly adherent to the surrounding cartilage matrix. Combining TEC with HA and beta-tricalcium phosphate has been studied in animal models and shown favorable outcomes of OL repairs with the HA combination demonstrating better results (Shimomura et al., 2014a, 2017).

Bioceramics encompass both osteoconductive and bioresorbable properties which are favorable in the scenario of an osteochondral repair. To increase the elastic modulus of bioceramics, polymers have been combined with them and have shown encouraging cartilage regenerative results (Xue et al., 2010; Lv and Yu, 2015). Bioactive ions such as lithium, manganese, zinc, and silicon have also been under recent study to improve the bioactivity of the implants and shown promising results (Deng et al., 2017, 2019).

### Subchondral Layer

As mentioned earlier the subchondral bone is responsible for providing compressive strength to the osteochondral unit and has a low elastic modulus. Currently available materials that meet this requirement include metals, bioglass, and bioceramics (Kon et al., 2010; Chen et al., 2011; Zhang et al., 2013; Deng et al., 2017). Metallic compounds are inert and therefore have been popular in orthopedic surgery, however, for integration they must possess a basic level of bioactivity. This led to coating metals with HA and calcium phosphate thereby promoting better implant integration but not addressing the degradation of the material. Magnesium base alloys are now being studied as they possess adequate mechanical strength, bioactivity, and degradation (Yang et al., 2018). Overall, wear particle release and corrosion remain a limitation when using metallic materials (Sonny Bal et al., 2010).

Ceramics and bioglass possess excellent osteoconductive and inductive properties which allow them to bond well to the adjacent host bone (Tamai et al., 2005; Martin et al., 2007). These materials are, however, brittle and can fracture under mechanical loading (Nooeaid et al., 2012; Deng et al., 2019). By modifying the porosity of ceramics, their biodegradability can be effectively altered and titrated to the desired rate. Porosity and mechanical strength are inversely related and the addition of biodegradable polymers can help solve this problem (Miao et al., 2008; Ren et al., 2008). The integration of these subchondral substitution materials with a chondral natural or synthetic polymers, e.g., collagen, hyaluronic acid, poly (glycolic acid) can together manufacture an osteochondral unit with materials that satasify the functions of both the chondral and subchondral layers.

## Clinical Results of Osteochondral Implants

Clinical data utilizing multiphasic osteochondral implants is sparse along with the fact that only three such osteochondral implants have been used. These are MaioRegen (Fin-Cermica Faenza SpA), TruFit (Smith and Nephew, Andover, MA, United States) and more recently Agili-C (Cartilheal Ltd, Kfar Sava, Israel). MaioRegen and TruFit have been studied and reported further than Agili- C and clinical trial results for Agili-C are awaited.

Recently, D’Ambrosi et al. (2019) published a systematic review on the results of MaioRegen. MaioRegen being a triphasic scaffold aims to closely resemble the osteochondral unit. 471 patients were included in the review with a mean follow up of 24 months. 15 out of the included 16 studies were level IV and only one was a comparative level III study. The included lesions were all ICRS grade III and IV excluding two studies which included spontaneous osteonecrosis of the knee and Kellgren- Lawrence grade III OA. Clinical outcome scores at 24 months demonstrated significant improvement in thirteen studies with only one study reporting no difference. Histological analysis was reported by only two studies and indicated no residual scaffold with a strong presence of type II collagen and proteoglycan content. This reveals that the implant resorption and regenerative tissue response is adequate. Complications included 2 partial implant detachments, 2 cases of graft hypertrophy, and 52 patients reported minor complications such as joint stiffness and swelling. There were 16 failures in this systematic review. On the whole, the results of MaioRegen have been favorable with good clinical results and a low complication rate, however, the included studies were of a low level of evidence and as a result, they could not conclude that MaioRegen was superior to other treatments till better randomized trials were performed.

TruFit is a biphasic acellular synthetic scaffold mainly composed of a polylactide-coglycolide copolymer for the chondral region and calcium sulfate for the bone region. TruFit has shown to have a clinical benefit at 12 months of follow up but two studies reported worsening on longer follow up as reported by a systematic review in 2015 (Verhaegen et al., 2015). The main complication reported with use of the TruFit implant was the failure of bony ingrowth and fissured lesions in the surface of the chondral regenerate at 24 months. Few studies reporting histology also showed the presence of subchondral cysts and fibrous cartilage, but it should be noted these were biopsies performed in patients that required revision (Dhollander et al., 2012; Joshi et al., 2012). TruFit being a synthetic scaffold appears to have issues with biodegradability and integration and therefore needs to be further improved before further clinical application (Verhaegen et al., 2015).

A more recent developed synthetic osteochondral implant is Agili-C which has a chondral phase made up of modified aragonite with hyaluronic acid and a bone phase of calcium carbonate. Only one clinical case report is published with most results of the Agili-C implant being in pre-clinical studies. Pre-clinical studies have shown excellent cell recruitment and biocompatibility of the materials (Kon et al., 2014, 2015; Chubinskaya et al., 2019). Kon et al recently reported the use of a hemicondylar aragonite implant in a caprine model. At 12 months follow up they found the implant promoted good chondral and subchondral regeneration, excellent integration and no adverse effects (Gomoll, 2020; Kon et al., 2020). In the clinical case study, a 47-year-old male patient underwent an osteochondral repair with Agili-C and reported significant improvement in functional outcome scores. Radiographic studies at 24 months indicated hyaline cartilage regeneration over the entire defect and good bone integration. Sequential radiography suggested the entire implant degraded and was substituted for cartilage and bone by creeping substitution. Results of the Agili-C implant are encouraging, and a randomized clinical trial has been underway. Hopefully, in the near future, these results will be available to better evaluate and make recommendation guidelines for the use of Agili-C Table 3. Summarizes the clinical studies using multiphasic scaffolds.

**TABLE 3:** Summarizes the clinical studies using multiphasic scaffolds.

**Author/Year**	**Type of study**	**Patient number**	**Implant specifics/company**	**First clinical trial**	**Lesion size/cm^2^**	**Follow up/months**	**Results**
D’Ambrosi et al., 2019	Systematic	471	MaioRegen	2011–2016	3.6 ± 0.85	24	Satisfactory mid-term follow-up results and
	review		Triphasic				quicker return to sports. Low complication and
			C: Coll I				failure rates.
			B:				
			70%HA-30% Coll I				
			30%HA-70%Coll I				
			/Finceramica, Italy				
Verhaegen et al., 2015	Systematic	130	TruFit	2010–1015	N/A	12	No evidence for TruFit being superior or equal
	review		Biphasic				to other techniques. Longer follow ups result in
			C: PGA, PLGA, surfactant				poorer outcomes. Subchondral integration is
			B: Ca Sulfate				inferior with bone cysts reported. Chondral
			/Smith & Nephew, United				regenerate contains fibroblastic tissue.
			States				
Kon et al., 2014	Case report	1	Agili-C	2015–2020	2	24	MRI: Good tissue integration. Regenerate
			Biphasic				resembled hyaline cartilage. VAS, Lysholm.
			C: Modified aragonite + HA				Tegner, IKDC scores significantly improved with
			B: Aragonite				excellent outcomes
			/CartiHeal (2009) Ltd, Israel				

## Future Direction

As several of the proposed strategies to treat OLs remains in experimental and pre-clinical phases, it is difficult to predict which will prove most useful in the clinical management of OLs. We see popular chondral substitutes being derived from polymers and ECMS while the subchondral materials frequently used are ceramics, bioglass, and metals. These materials seeded with stem cells, growth factors, different culture methods or bioactive ions show encouraging results with the most effective combination of these yet to be determined. Clinical recommendations for the use of osteochondral implants are awaited pending further well-designed trials. The present literature reports encouraging results but in the interim osteochondral fragment fixation, OATs and OCA remain techniques with respectable outcomes so long as their specific indications and limitations are noted.

## Author Contributions

GJ wrote the first draft of the manuscript. KS and NN edited the manuscript. All authors contributed to manuscript revision, read and approved the submitted version.

## Conflict of Interest

The authors declare that the research was conducted in the absence of any commercial or financial relationships that could be construed as a potential conflict of interest.
